# Talimogene Laherparepvec: Moving From First-In-Class to Best-In-Class

**DOI:** 10.3389/fmolb.2022.834841

**Published:** 2022-02-22

**Authors:** Howard L. Kaufman, Sophia Z. Shalhout, Gail Iodice

**Affiliations:** ^1^ Division of Gastrointestinal and Oncologic Surgery, Department of Surgery, Massachusetts General Hospital, Boston, MA, United States; ^2^ Ankyra Therapeutics, Boston, MA, United States; ^3^ Division of Hematology/Oncology, Department of Medicine, Massachusetts General Hospital, Boston, MA, United States

**Keywords:** biomarker, cancer, immunotherapy, oncolytic virus (OV), treatment

## Abstract

Talimogene laherparepvec (T-VEC) is a modified oncolytic herpes Simplex virus, type 1 (HSV-1) encoding granulocyte-macrophage colony stimulating factor (GM-CSF). T-VEC is adapted for selective replication in melanoma cells and GM-CSF was expressed to augment host anti-tumor immunity. T-VEC is indicated for the local treatment of melanoma recurrent after primary surgery and is the first-in-class oncolytic virus to achieve approval by the FDA in 2015. This review will describe the progress made in advancing T-VEC to the most appropriate melanoma patients, expansion to patients with non-melanoma cancers and clinical trial results of T-VEC combination studies. Further, strategies to identify predictive biomarkers of therapeutic response to T-VEC will be discussed. Finally, a brief outline of high-priority future directions for investigation of T-VEC and other promising oncolytic viruses will set the stage for a best-in-class oncolytic virus to bring the maximum benefit of this emerging class of anti-cancer agents to patients with cancer.

## Introduction

Oncolytic viruses (OV) are a new class of anti-cancer therapeutic agents that utilize native or genetically modified viruses to treat cancer. While early reports of tumor regression in patients infected with various viruses has been reports for over a century, advances in molecular genetics and virology have only recently allowed a more directed approach to therapeutic development in this area (Kucerova and Cervinkova, 2016). Based on these early observations, it was thought that OVs most likely mediated tumor regression by preferentially infected and killing tumor cells. Indeed, many cancer cells express high levels of viral entry receptors, and recent data demonstrating defects in the anti-viral machinery in cancer cells, provides a logical mechanism for selective tumor cell killing by oncolytic viruses (Kaufman et al., 2015). Some native viruses possess innate oncolytic activity, and functional tumor cell killing may be enhanced by serial passage through specific cancer cells selecting for viral clades with the highest lytic potential. Alternatively, many viruses can be genetically engineered through deletion or insertion of various viral genes designed to enhance tumor selective replication (Kaufman et al., 2015). While direct viral-mediated lysis of cancer cells was widely accepted as an important process for OV-induced anti-cancer activity, this now appears to not be the major mechanism of action for most OVs.

Viruses are among the most immunogenic agents recognized by the host immune system and the ability of OVs to induce immune responses likely explains the major mechanism involved in OV-mediated anti-cancer activity (Harrington et al., 2019). The induction of host immune responses against viral antigens is dependent on recognition of viral peptides within infected host cells, and this process allows cancer cells to be specifically targeted for T cell-mediated effector functions (Milich, 1987). This process, which has been referred to as immunogenic cell death allows tumor-associated antigens to be released in the context of an active viral infection, which releases danger-associated factors, that results in immune recognition and eradication of OV-infected cells. In addition, release of soluble tumor antigens can also result in antigen spreading and this allows immune recognition, and in some cases eradication, of non-infected cells. This has been described as an “abscopal” or “anamnestic” response (Andtbacka et al., 2016a). Animal models have confirmed that injection of an index tumor can cause regression of uninjected tumors in an immune-dependent manner (Zamarin et al., 2014). Thus, OVs provide two independent mechanisms that can reinforce tumor-specific immune clearance.

Progress in molecular biology and cloning technology have also allowed expression of eukaryotic genes by viruses. Genomic stability and expression levels are dependent on the size of the gene or genes expressed, the size of the viral genome, the impact on viral integrity and likely additional epigenetic factors, large viruses have been shown to efficiently encode multiple human genes, which can be used to provide additional anti-tumor activity. In many cases, the genes selected for expression are cytokines to enhance local immune responses against the virally infected cancer cells, other strategies have included expression of suicide genes, apoptosis-inducing genes, and radiosensitizers among others (Kaufman et al., 2015). The contribution of transgene expression has not been fully elucidated but does offer an additional pathway for optimizing anti-tumor immunity and therapeutic responses.

While OVs have demonstrated proof-of-principle in a multitude of pre-clinical tumor models, clinical development has been slower. Globally, four OVs have been approved for cancer therapy. In the People’s Republic of China, an oncolytic E1B-deleted adenovirus (H101; Oncorine^®^) is approved in combination with chemotherapy for treatment of head and neck cancers (Liang, 2018). An unmodified picornavirus (enterovirus, ECHO group, type 7; Rigvir^®^) is approved for the treatment of melanoma in several Eastern European countries (Alberts et al., 2018). In November 2021 a triple-mutated oncolytic HSV-1 (G47Δ), teserpaturev (Delytact), was approved in Japan for the treatment of malignant glioma (Nguyen and Saha, 2021). The only OV to achieve approval in the United States is Talimogene laherparepvec (T-VEC; Imlygic^®^), which was granted U.S. Food and Drug Administration (FDA) approval in 2015 for the treatment of melanoma (Andtbacka et al., 2015). T-VEC has subsequently been approved throughout Europe, and in Australia and Israel. Since approval, we have learned a lot about both the challenges and best clinical indications for T-VEC treatment. This review will describe the initial clinical development of T-VEC and then focus on our current understanding based on both real-world experience and new clinical trials with T-VEC. While T-VEC has provided another option for patients with melanoma, the integration of T-VEC into clinical practice occurred at a time of unprecedented therapeutic advances in melanoma, including the approval of BRAF/EK targeted therapy and single agents as well as combination immune checkpoint blockade (Luke et al., 2017). Nonetheless, the potential role for OVs, such as T-VEC, remains intriguing and a high priority for predictive biomarkers is needed to better select appropriate patients for effective therapy while avoiding potential toxicities. We will mention some recent insights into biomarkers of OV responses and complete the review by discussing anticipated future directions for T-VEC and other OVs in clinical development.

## The Development of Talimogene Laherparepvec for Melanoma

T-VEC is based on a modified oncolytic herpes Simplex virus, type 1 (HSV-1) that was originally isolated from a fever blister (Liu et al., 2003). The virus was selected for *in vitro* oncolytic activity against a range of tumor cell lines and further modified by deletion of the two viral infected cell protein (ICP) 34.5 genes, which encodes the neurovirulence factor and deletion improves tumor cell selective replication. In addition, the viral ICP47 gene is deleted and this encodes a viral inhibitor of peptide attachment to major histocompatibility complex (MHC) class I, which the virus uses to prevent immune detection during natural infections. The ICP47 deletion was thought to be important in allowing MHC class I loading of tumor-associated peptides, which would be necessary to promote anti-tumor immunity. Finally, T-VEC is modified by inserting two copies of the human granulocyte-macrophage colony stimulating factor (GM-CSF) genes to promote dendritic cell recruitment and activation following antigen uptake from lysing tumor cells. The virus demonstrated therapeutic activity in the murine A20 lymphoma model and was adapted for clinical translation (Liu et al., 2003).

The first clinical trial of T-VEC was reported in 2006 in a phase I study of 13 patients with a variety of cancers, including melanoma, breast, head and neck and gastrointestinal tumors (Hu et al., 2006). Virus was given by direct intra-tumoral injection into superficial, subcutaneous, or nodal accessible tumors. This study established the safety profile, which included low grade constitutional symptoms, such as fatigue, fevers, chills and nausea, and local injection site reactions. Biopsy of injected tumor sites revealed necrosis and other signs of inflammation with virus found only in viable tumor cells and evidence of local GM-CSF expression was confirmed. A series of dosing schedules was used and toxicity was generally lower in patients treated with a lower priming does of 1 × 10^6^ plaque-forming units (pfu) to allow seroconversion in HSV-1-naïve patients, followed by a higher dose of 1 × 10^8^ pfu. This was followed by a multi-institutional phase 2 single-arm study of T-VEC in patients with superficially accessible melanoma (Senzer et al., 2009). In this study an objective response rate of 26% was observed and the safety profile was similar to the profile seen in the phase I study.

Based on the emerging data from the early phase clinical trials, the OPTiM study, was developed as a prospective, multi-institutional randomized phase III clinical trial to determine the clinical benefit of T-VEC in patients with superficially accessible melanoma. In this study, 436 patients with stage IIIB-IV melanoma were randomized in a 2:1 manner to treatment with T-VEC or recombinant GM-CSF. The control arm was selected to allow study participants to receive potentially active therapy and at the time there was interest in single agent GM-CSF for melanoma, although this was not supported by subsequent studies (Spitler et al., 2000). The study used a primary endpoint of durable response rate that was defined as an objective response per modified World Health Organization (WHO) criteria and persistent for at least 6 months. An objective response rate of 26.4% was seen compared to 5.7% for patients treated with GM-CSF; and durable response was 16.3% compared to 2.1%, which met the primary study endpoint. In addition, median overall survival was improved in T-VEC-treated patients compared to GM-CSF therapy [23.2 vs. 18.9 months (hazard ratio, 0.79; 95% CI, 0.62 to 1.00; *p* = .051)] (Andtbacka et al., 2015). At a median follow-up of 49 months, a final analysis was performed and demonstrated an objective response rate of 31.5 versus 6.4% for GM-CSF was reported and durable response rate of 19% for T-VEC vs. 1.9% for GM-CSF (*p* < .0001) was seen (Andtbacka et al., 2019). Further, the OS benefit for T-VEC compared to GM-CSF persisted (hazard ratio, 0.79; 95% CI 0.62, 1.00; *p* = .0494). The 5-year survival for patients was 33.4% and the impact on survival was most pronounced for patients with stage IIIB/C and IVM1a melanoma (hazard ratio, 0.57; 95% CI 0.41, 0.81; *p* < .001). In the OPTiM trial, 54% of patients exhibited some degree of disease progression based on caliper measurement or imaging prior to achieving an objective response, suggesting that pseudo-progression may be possible with T-VEC treatment. Based on these data T-VEC was approved by the U.S. FDA in 2015 for the local treatment of melanoma that recurs after initial surgery and T-VEC was approved in Europe in 2016 for the local treatment of melanoma patients with stage IIIB-IVM1a disease. Australia, Israel, and Switzerland have also approved T-VEC for the treatment of melanoma. The adverse events in the phase III clinical trial were similar to earlier phase studies establishing a favorable safety profile for T-VEC.

T-VEC was the first OV approved for cancer treatment and provided a new therapeutic strategy for patients with melanoma. Importantly, the approval in 2015–2016 corresponded to a time with major changes in the therapeutic landscape of melanoma. In 2011 the first BRAF inhibitor was approved for metastatic melanoma patients with BRAF V600E/K mutated tumors, which would be followed by combination BRAF and MEK inhibition therapy (Chapman et al., 2011; Flaherty et al., 2012). In addition, immune checkpoint inhibition achieved approval initially with ipilimumab, a monoclonal antibody that blocks the cytotoxic T lymphocyte antigen 4 (CTLA-4) negative T cell regulatory, in 2011 and then with pembrolizumab and nivolumab, both monoclonal antibodies that block the programmed cell death 1 (PD-1) checkpoint, in 2015. Thus, multiple new drugs in the targeted therapy and immunotherapy arena became accessible for melanoma patients. These drugs would also go on to be approved in the adjuvant setting (Eggermont and Dummer, 2017). Thus, the clinical implementation of T-VEC would take some time to integrate with other agents available for the treatment of advanced melanoma.

## Real World Experience With T-VEC

**FIGURE 1:**
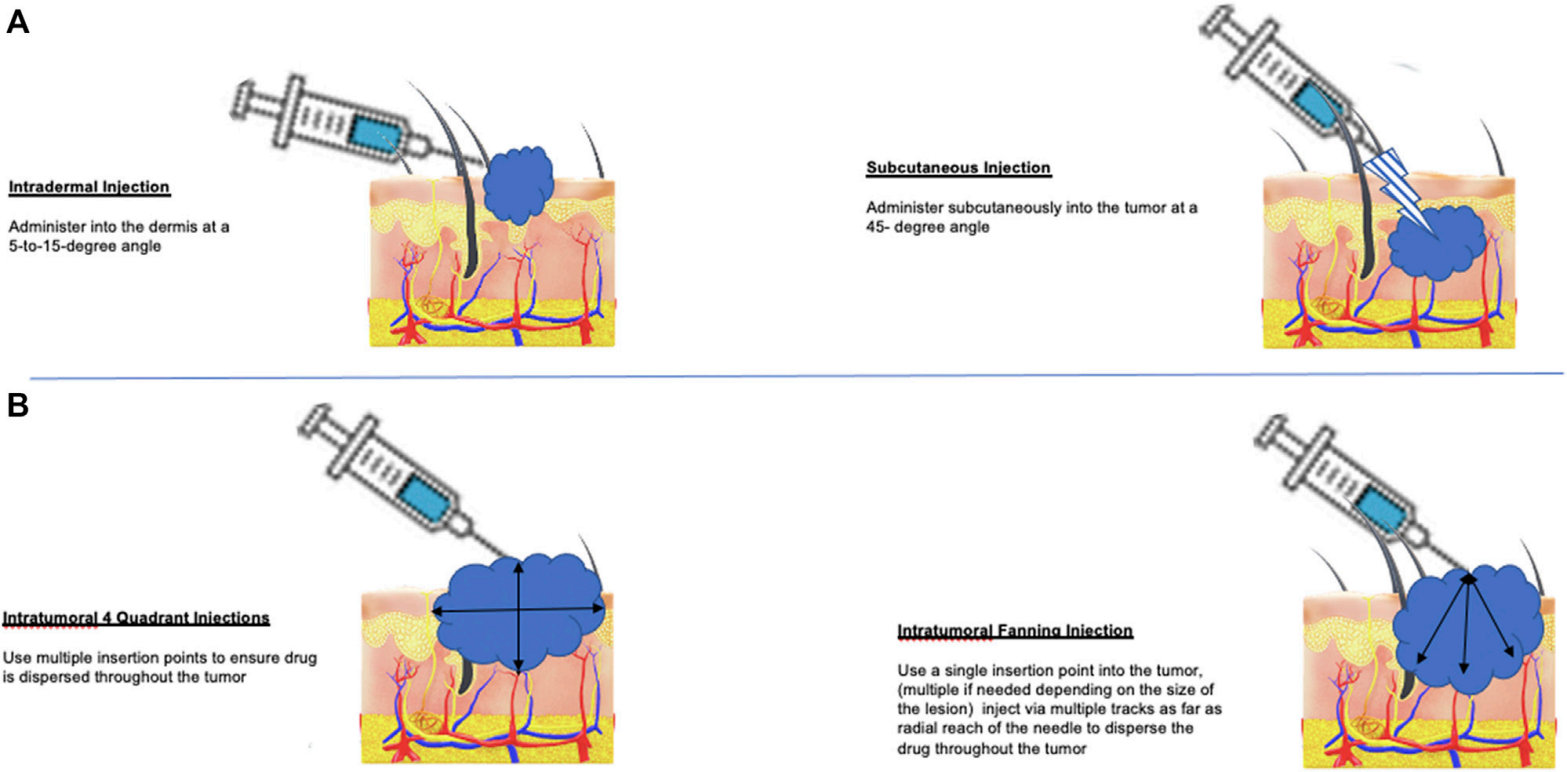
Methods for T-VEC administration in patients with melanoma. **(A)** T-VEC can be administered by intradermal injection (left panel) at sites of cutaneous tumor or by subcutaneous injection (right panel) for tumors in the soft tissue or lymph nodes. If tumors are not clinically palpable or become undetectable after initiating treatment, portable ultrasound can be used to identify residual areas of tumor for injection. **(B)** The goal of injection is to distribute the virus as evenly as possible throughout areas of viable tumor cells. This can be done using a four-quadrant method (left panel), which allows re-insertion to reach all sites and may be preferred for large tumors; alternatively, a single injection site and then using a fan technique (right panel) for injection can also be used. In tumors with necrotic or liquid areas, peripheral injection at the edges where most viable tumor cells are located is also acceptable.

The launch of T-VEC was challenging for several reasons. First, OV storage and intratumoral delivery of the agent offered unique challenges for local pharmacies and healthcare providers, as outlined in Table 1. In addition, T-VEC was approved around the same time as the approval of immune checkpoint blockade and targeted therapy, which are given by intravenous and oral administration, respectively. Because several other promising agents were available, many patients were considered for T-VEC only after having progressive disease after other treatments. Over the last 5 years, however, there has been considerable real-world data published providing a better idea of how best to integrate T-VEC treatment into the clinical practice and how to optimize patient selection and management (Perez et al., 2018; Louie et al., 2019; Mohr et al., 2019; Perez et al., 2019; Louie et al., 2020; Sun et al., 2020; Kleemann et al., 2021; van Akkooi et al., 2021). In addition, techniques for injecting T-VEC have now been well established (Figure 1).

**TABLE 1:** Barriers and challenges to oncolytic virus clinical implementation.

Challenge to OV implementation	Comments
Requires storage at −80°C	• Many pharmacies do not have deep freezer capabilities
Live virus must be prepared in sterile biosafety cabinet	• Dedicated preparation space is often difficult in pharmacies preparing chemotherapy and other agents
	• Contamination of other drug products requires strict SOPs and dedicated time, space, and training for pharmacists
Drug dosing is different for initial injection vs. later timepoints	• Two different doses must be maintained and prepared appropriately
Drug volume is dependent on maximal tumor diameter	• Volume cannot be determined until the patient has tumor measured resulting in ordering delays and longer patient treatment wait times
	• May require new ordering forms/processes
Injection requires direct access to tumor site and manual administration	• Lesions may not be palpable or may regress to a size that is not detectable
	• Bedside ultrasound can help guide injections and may be used when lesion regress below levels of clinical detection
	• Technical training is required for optimal delivery
	• May be administered by non-physicians, such as nurse practitioners or physician assistants
Biosafety concerns	• OV are typically live, replicating viruses and clinics must adopt biosafety measures for spills and waste
	• Usually only requires standard universal precautions
Household and healthcare transmission	• Virus can be transmitted to close contacts
	• Acyclovir and other anti-virals which may be used in cases of inadvertent exposure
	• Transmission can be prevented by barrier bandages and educating patients to avoid direct contact between injection site and other individuals
	• Training for healthcare providers, affiliated clinic staff, patients and patient families may help prevent accidental spread
May require change to ambulatory practice	• Can improve process by dedicating specific room(s) and clinic day(s) for OV injection
	• Healthcare centers may require written SOPs and approval by biosafety and/or infection control committees
	• Practice deviations may be difficult if only a limited number of patients are treated with OV therapy at site

In a retrospective review of T-VEC in patients with stage IIIB-IV1a melanoma at a single German facility, 27 patients were treated with T-VEC between June 2016 and July 2017 (Mohr et al., 2019). All patients had undergone prior surgery and 63% of the patients received T-VEC as their first line of melanoma treatment. Of these patients, only one required subsequent systemic treatment. In a separate, multi-institutional U.S.-based retrospective review, 121 patients receiving T-VEC from October 2015 through October 2018 were identified with a median follow-up of 9 months and 80 patients were available for evaluation (Louie et al., 2019). Thirty-four (42.5%) of the patients evaluated received T-VEC as first-line treatment and an objective response was seen in 45 (57%) of patients after a median of six treatment cycles, including a complete response in 31 (39%) and partial response in 14 (18%) of patients, higher than that observed in the OPTiM phase III clinical trial (Andtbacka et al., 2015). In another independent review of T-VEC treatment at seven academic institutions, 76 patients were identified over a similar time period as these other trials but included a substantial number (43.4%) who had received prior checkpoint blockade prior to T-VEC treatment (Perez et al., 2019). Fifteen (19.7%) of patients achieved a pathologic complete response to treatment after a median duration of 3 months of treatment. Importantly, all these studies confirmed the initial safety profile of T-VEC and found that therapy was generally well tolerated with mostly low-grade constitutional symptoms and local injection site reactions. Collectively, these studies suggested that early use of T-VEC in the first-line setting may be preferrable to more advanced clinical settings.

A more recent real-world report was published on 127 patients in the national German prescription database (Louie et al., 2020). Of the patients identified, two-thirds were started in or after 2017 and most (88%) were treated by hospital sites. At the end of the study, 26 (36%) of the patients remained on T-VEC and the overall median duration of treatment was 18.7 weeks and was longer for those who started treatment in 2017 compared to those treated in 2016 (26.7 vs. 15.6 weeks, respectively. The authors concluded that with more clinical experience after 2017, patients were appropriately kept on treatment longer as more physicians recognized the possibility of pseudo-progression following T-VEC treatment. Another trial reported for patients in the 2017–2018 era evaluated the relationship between T-VEC and anti-PD-1 treatment (Sun et al., 2020). In 83 patients from multiple institutions, three patterns were observed. Twenty-two (26.5%) of the patients received T-VEC after anti-PD-1 therapy, 32 (38.6%) received T-VEC concurrently with anti-PD-1 therapy, and 29 (34.9%) of patients only received T-VEC. Across all groups the objective response rate was 25% and the authors concluded that T-VEC could be used in combination with checkpoint blockade and sequencing did not appear to influence therapeutic responses.

In another single institution retrospective study of T-VEC, 27 patients with a median age of 75 years were treated and results reported at a median follow-up of 8.6 months (Perez et al., 2018). In this study most patients had prior therapy, including four patients having isolated limb perfusion, five patients having prior systemic immunotherapy and four patients having both prior to T-VEC. Further, 22 (81.5%) of the patients had stage III disease and five patients (18.5%) had stage IV disease at the time of T-VEC treatment. Of the 27 patients, 23 met criteria for response assessment and there was disease control rate of 78.3% reported with ten patients (43.5%) having a complete response. The authors concluded that there is a high response to T-VEC and upfront selection of patients with limited disease burden, such as in-transit metastases, may be helpful in improving the likelihood of response.

In a small study of 12 melanoma patients with a median age of 83 years, T-VEC was used and resulted in an overall response rate of 58.3%, durable response rate of 41.7% and a complete remission rate of 25% (Kleemann et al., 2021). In this cohort there were no grade 3 or higher treatment-related adverse events noted. The authors concluded that T-VEC may be an important consideration for older patients with melanoma who may not be able to tolerate other systemic options. Overall, the real-world data suggests that objective responses and safety profile for T-VEC in melanoma patients are comparable to those observed in the OPTiM phase III clinical trials (van Akkooi et al., 2021). Clinical benefit may be especially high in older patients and in those receiving T-VEC as first-line treatment. Although these studies are subject to bias due to their retrospective nature and influenced by treatment changes in both adjuvant and metastatic melanoma therapy over time, they do support the concept of using T-VEC earlier in the disease course and that T-VEC may be a safe option for older patients who may not be eligible for other systemic treatments.

## Expanding the Clinical Indications for T-VEC in Melanoma

In a subset analysis of the OPTiM clinical trial, a higher response rate was noted in patients with head and neck melanomas (Andtbacka et al., 2016b). Of the 436 patients enrolled in the phase III randomized trial, 87 (19.9%) had melanomas located in a head or neck location. Of these 87 patients, 61 were treated with T-VEC and 26 with recombinant GM-CSF. The durable response rate was 36.1% for patients treated with T-VEC compared to 3.8% for GM-CSF and 16.3% for all patients treated with T-VEC). A complete response was seen in 29.5% of the head and neck melanoma patients treated with T-VEC. The probability of maintaining an objective response after 12 months was 73%. While the overall survival of the entire T-VEC-treated population was 23.2 months, the median overall survival had not been reached in the head and neck melanoma subset. While it is tempting to hypothesize that the head and neck melanomas may be more responsive due to the increased tumor mutation burden likely related to Sun exposure, this has not been formally confirmed. Nonetheless, the data suggests that there may be subsets of melanoma patients more likely to benefit from T-VEC treatment.

Another subset of melanoma patients that were not treated in the OPTiM trial are patients with organ allografts. This represents an important unmet medical need as malignancy is more common in transplant recipients with an increased incidence over time and cutaneous tumors, including melanoma are especially common. Because of the risk of allograft rejection, treatment with potent immunotherapy, such as immune checkpoint blockade, may not be possible. Thus, the potential benefit of OV therapy in this setting has gained recent attention. Indeed, there are two case reports demonstrating complete responses of locally advanced melanoma with T-VEC treatment in transplant recipients, one with a heart transplant and one with a heart and kidney transplant (Schvartsman et al., 2017; Ressler et al., 2019). In both cases, no new safety signals were reported. Further clinical studies are needed to better understand the full risk-benefit potential for T-VEC and other OVs in patients with transplant-related melanoma.

Although T-VEC was originally developed for patients with advanced melanoma. It is well suited for earlier use as, for example, in the neoadjuvant setting. The rationale for this is to provide an opportunity for T-VEC to induce host anti-tumor immunity by using established tumors as a source for *in situ* vaccination at an earlier time prior to extensive immunoediting as occurs in metastatic disease. A randomized phase 2 trial was conducted in 150 patients with resectable stage IIIB-IVM1a melanoma (Dummer et al., 2021). In this study 76 patients were randomized to six doses of T-VEC followed by surgery and 74 patients received surgery alone with a primary endpoint of 2-year relapse-free survival (RFS) in the intention-to-treat population. The 2-year RFS was 29.5% in the T-VEC arm and 16.5% in the surgery alone arm (overall hazard ratio 0.75, 80% CI, 0.58–0.96). In addition, 2-year overall survival was improved in patients treated with T-VEC followed by surgery compared to surgery alone (88.9 vs. 77.4%; overall hazard ratio 0.49, 80% CI, 0.30–0.79). The pathologic complete response rate in patients treated with T-VEC was 17.1%). This data is promising but requires larger sample size and longer follow-up to better define the true benefit of neoadjuvant T-VEC for melanoma. Given the high-risk for recurrence associated with some subsets of early stage I-II melanoma patients, successful demonstration of a neoadjuvant benefit could also help support clinical studies of T-VEC in high-risk stage II melanoma.

## Expanding T-VEC to Other Cancers

While T-VEC was approved for the treatment of melanoma, the virus was able to demonstrate activity against tumor cells derived from other histologic tumors *in vitro* (Liu et al., 2003). This suggests that the agents may be useful in other types of human cancer. This is a concept that has been evaluated now in a small number of clinical trials with interesting yet inconclusive results. In general, accessible tumors for intratumoral injection have been a priority, and this has included head and neck squamous cell carcinoma, soft tissue sarcoma and breast cancer.

A small trial of 17 patients with stage III or IV squamous cell carcinoma of the head and neck was conducted with T-VEC in combination with *cis*-platinum chemotherapy and radiation therapy followed by surgery (Harrington et al., 2010). Fourteen (82.3%) of patients demonstrated objective responses by imaging or clinical exam with 93% showing pathologic complete response at the time of surgery. At a median follow-up of 29 months, disease-specific survival was seen in 82.4% of patients. Although the number was small, the results supported further studies in head and neck cancer. In a phase Ib/III multi-institutional clinical study, T-VEC was evaluated in combination with pembrolizumab in patients with recurrent or metastatic head and neck squamous cell carcinoma (Harrington et al., 2020). Thirty-six patients were entered into the phase Ib portion of the study and data was reported at a median follow-up of 5.8 months. Ten patients (27.8%) of the patients were not evaluable for response due to early mortality. A confirmed partial response occurred in 5 (13.9%) of patients. The authors concluded that while treatment was generally well tolerated combination therapy was not associated with a benefit compared to historical studies with pembrolizumab alone and the phase III portion was not pursued further. This trial only administered T-VEC into cutaneous, subcutaneous, and nodal tumor but did not allow injection into mucosal or visceral sites of disease.

Soft tissue sarcomas have also been targeted for clinical study of T-VEC with intriguing results to date. An open-label, single institution, phase 2 study of T-VEC and pembrolizumab was conducted in 20 patients with locally advanced or metastatic sarcoma patients who had disease progression after at least one other systemic therapy (Kelly et al., 2020). The study was designed with a primary endpoint of objective response rate at 24 weeks and all 20 patients were evaluable for response. The overall objective response rate was 35 and 20% of patients experience grade 3 treatment-related adverse events although no grade 4 events were seen. The authors concluded that the combination of T-VEC and pembrolizumab was worthy of further evaluation. In addition, another phase Ib/II study of T-VEC administered with standard pre-operative external beam radiation therapy was evaluated in patients with locally advanced soft tissue sarcomas of the trunk and extremities measuring more than 5 cm and for whom neoadjuvant radiation therapy was indicated (Monga et al., 2021). In this trial, one patient with a myxoid liposarcoma demonstrated a partial response and 7 (24%) patients had a 95% pathologic necrosis seen in resected tumor. The authors reported no dose-limiting toxicity and no patients had evidence of local recurrence after surgery. The 2-year overall survival was 88% and progression-free survival was 57%. The authors concluded the combination of T-VEC and pre-operative radiation was safe and further studies were warranted.

Breast cancer is another tumor that has been targeted for treatment with T-VEC since recurrent tumors are often accessible for direct injection. T-VEC was evaluated as a strategy for enhancing response to neoadjuvant chemotherapy in patients with triple-negative breast cancer (Soliman et al., 2021). In this phase I clinical trial, nine patients were treated with T-VEC at two dose levels in combination with paclitaxel followed by doxorubicin and cyclophosphamide for 8 weeks prior to surgery. The primary endpoint of the study was safety and no dose-limiting toxicities were reported. A complete pathologic response was seen in 55% of patients. In another phase 2 study, T-VEC was tested in breast cancer patients with inoperable locoregional recurrence (Kai et al., 2021). Nine patients were enrolled and six patients had locoregional disease only and three had additional metastatic lesions. While no significant adverse events were reported, no patients had an objective response. The authors suggested that further studies should consider combination approaches. A study of T-VEC in combination with atezolizumab, an anti-PD-L1 agent, in patients with operable HER2-negative breast cancer with residual disease after neoadjuvant chemotherapy is planned (Pascual et al., 2020). Other studies have been conducted to evaluate T-VEC in pancreatic cancer, hepatocellular carcinoma and non-melanoma skin cancers (NCT00402025; NCT02509507; NCT04163952).

## Exploring T-VEC Combination Strategies

T-VEC infection triggers type 1 interferon production by infected cells, and this in turn can result in expression of immune inhibitory surface receptors, such as PD-1 ligand 1 (PD-L1) on tumor cells (Bommareddy et al., 2018). Even when interferon signaling in tumor cells is defective, local infection of normal cells can drive local interferon production and, it is now clear, PD-L1 expression can inhibit viral clearance and may also be associated with suppressed immune clearance of tumors. Thus, it is logical to combine T-VEC with immune checkpoint blockade to enhance anti-tumor immunity (Ribas et al., 2017). In a small phase I clinical trial T-VEC and pembrolizumab demonstrated a 62% objective response rate in melanoma patients (Ribas et al., 2017). Further, this study demonstrated that T-VEC was able to induce regression of lymphocyte-deficient tumors, which is a negative predictive feature of pembrolizumab responses. The high response rate observed was the impetus for a larger, prospective randomized phase III trial of T-VEC and pembrolizumab versus placebo and pembrolizumab (Gogas et al., 2021). Unfortunately, after enrolling 692 patients in this global clinical trial, no benefit was observed for the combination treatment. The combination group had an overall response rate of 48.6% compared to 41.3% for pembrolizumab alone, which was not statistically significant Furthermore, the median OS was also not different between treatment arms with a median of 49.2 months for pembrolizumab alone and it was not reached for the combination treatment arm (hazard ratio 0.96, 95% CI 0.76, 1.24, *p* = .74). The reasons for the lack of benefit are not entirely clear as the final data has not yet been published. It is possible that the response rate of pembrolizumab alone in stage IIIB-IVM1a melanoma is higher than in stage IV disease and the study was not adequately powered to detect a narrower response difference between arms. Another difference between the phase I and III trial was that in the phase I study pembrolizumab was started after the second injection of T-VEC to allow seroconversion for HSV-naïve patients and avoid rapid vial clearance by enhanced anti-viral immune responses mediated by pembrolizumab. In the phase III trial, however, no T-VEC lead in was employed and both drugs were given on the first day. Further scrutiny of the data may be needed to better understand why this study was negative.

Interestingly, in another phase I study T-VEC was combined with ipilimumab, an anti-CTLA-4 monoclonal antibody in patients with advanced melanoma who were immune checkpoint inhibitor naïve (Puzanov et al., 2016). In this study a 50% response rate was seen with an acceptable safety profile comparable to adverse events seen with individual monotherapy. This was followed by a larger randomized phase II clinical trial in which 198 treatment-naïve melanoma patients were randomized to treatment with T-VEC and ipilimumab or ipilimumab alone (Chesney et al., 2018). The primary endpoint was objective response rate, which was more than doubled in the combination treatment arm (39 vs. 18%). In this trial, regression of un-injected visceral lesions was also seen in 52% of T-VEC and ipilimumab treated patients (vs. 23% with ipilimumab alone). The study met its primary endpoint but was not pursued for registration. A limitation of this study was that eligible patients were not allowed prior anti-PD-1 treatment, and thus, the therapeutic effectiveness of the combination in patients who have progressed after anti-PD-1 treatment is unknown.

## Predictive Biomarkers for Oncolytic Virus Responses

Predictive biomarkers of immunotherapy response have been important for better identifying patient populations likely to respond to treatment. For immune checkpoint blockade, several biomarkers are now recognized as clinically important, including a high tumor mutation burden, elevated local tumor PD-L1 expression, presence of tumor-infiltrating lymphocytes, and a high interferon gene expression pattern, all of which are associated with improved therapeutic responses (Cristescu et al., 2018). Biomarkers of OV response, however, have not been as well investigated but there are some new insights that have emerged from genomic studies of melanoma tumor cells.

**FIGURE 2:**
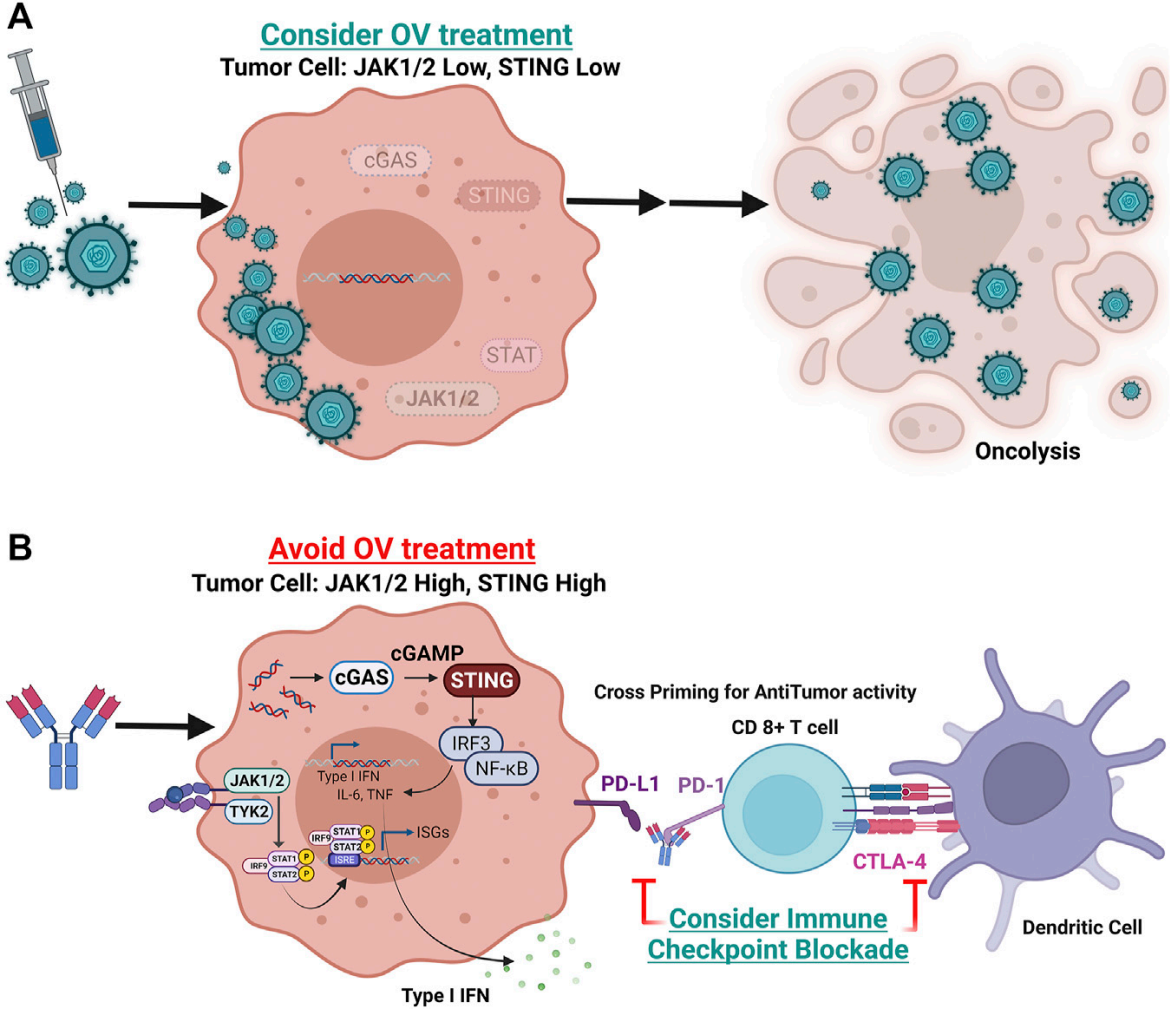
Potential predictive biomarker strategy for oncolytic virus therapeutic response. Shown are tumor cells with variable gene expression and interferon pathway signaling status at the time of initial diagnosis or pre-treatment biopsy. **(A)** Tumor cell with loss of function or low levels of JAK1, JAK2, and/or STING expression. In these tumors, oncolytic viruses may replicate more efficiently and induce widespread immunogenic cell death (oncolysis). **(B)** Tumor cells with intact JAK1, JAK2 and STING signaling will be resistant to DNA viral replication but are more sensitive to immune checkpoint blockade. These tumor cells will express higher levels of PD-L1 making them permissive to PD-1 blockade and potentially other checkpoint blockade, such as CTLA-4 (Created with Biorender).

In an intriguing study by Nguyen et al., next-generation sequencing and CRISPR-Cas9 screens identified mutations in the interferon-JAK-STAT signaling pathway in melanoma cells as associated with resistance to anti-PD-1 therapy (Nguyen et al., 2021). This study found a melanoma patient with disease progression after treatment with anti-PD-1 had mutations resulting in JAK1 and JAK2 loss of function. They showed that tumor cells without JAK1/JAK2 function, while resistant to anti-PD-1, were much more sensitive to OV infection. They also showed that genetic and pharmacologic inhibition of JAK function could enhance the oncolytic activity of OVs *in vitro*. These data suggest that JAK1 and JAK2 expression may be an important biomarker of OV activity but clinical validation is still required. In our lab we also found that loss of STING expression, a known biomarker for anti-PD-1 resistance, was associated with improved oncolytic activity of T-VEC *in vitro* (Bommareddy et al., 2019). Furthermore, low STING-expressing melanoma cells resistant to PD-1 blockade *in vivo*, were sensitive to T-VEC treatment supporting a role for STING expression as a biomarker of T-VEC response. Collectively, these data support a role for elements of the interferon signaling anti-viral machinery in tumor cells as possible predictive biomarkers of OV activity and merits further clinical investigation (Figure 2).

In addition to intracellular anti-viral machinery factors, other potential predictive biomarkers might include viral cell entry receptor expression on tumor and other stromal cells within the tumor microenvironment, high tumor mutation burden, high levels of tumor-infiltrating effector CD8^+^ T cells, low levels of regulatory CD4^+^ T cells, and the status of macrophage and myeloid-derived suppressor dendritic cells. In addition, there has been limited data on the association of anti-viral humoral and cellular immune responses with clinical outcome in OV clinical trials. Furthermore, metabolic, and nutritional factors, including the individual patient microbiome status, may impact viral infection and potentially OV-mediated anti-tumor therapeutic responses. Investigators should consider incorporating these biomarkers in future OV clinical trials to obtain exploratory data to identify those markers worth further prospective validation.

## Conclusion and Further Directions

T-VEC was the first-in-class OV approved for the treatment of melanoma. While treatment was initially approved for patients with melanoma that recurs after initial surgery, further real-world data has helped to better define which patients to treat and how best to implement T-VEC therapy in the ambulatory setting. The potential for objective responses is optimal when T-VEC is used in first-line therapy for locally and regionally advanced melanoma. As such, T-VEC should be considered early in the management of recurrent melanoma when surgical management may be technically feasible but is not considered curative, such as for management of in-transit melanoma metastases. In addition, an important aspect of T-VEC treatment is the potential for pseudo-progression, which occurs when tumors appear to increase in size or number by clinical exam or radiologic imaging but the increase is due to local inflammatory changes and not tumor progression. This has been seen with T-VEC alone and in combination approaches (Andtbacka et al., 2015; Chesney et al., 2019). Since the mean time to response in the phase III OPTiM trial was 4.1 months, it may be prudent to use immune related RECIST criteria or allow treatment past progression provided there no deterioration in clinical performance status. If there is uncertainty about the response, biopsy of the lesion can often resolve tumor progression or inflammation with regression. An outline of considerations in patient selection and treatment of patients in the clinic is shown in Table 2. The volume of T-VEC is based on the longest diameter of accessible tumors when patients present for treatment according to Table 3. Post-injection management pearls are provided in Table 4.

**TABLE 2:** Considerations for treating patients with T-VEC in the ambulatory setting.

• Establish institutional standard operating procedures
• Consider dedicating a single room and day for T-VEC treatment
• Provide education for healthcare providers handling T-VEC
• Before placing orders, measure the diameter of all tumors at each visit with calipers
• Select index lesions for injection (prioritize large > small size lesions; new > old lesions; avoid lesions near critical anatomic structures, e.g., carotid artery, mucosal surface)
• Use schema in Table 3 to determine volume
• NOTE: the maximum volume at any visit is 4 ml
• Ensure first dose is 10^6^ pfu/ml
• Ensure subsequent doses are 10^8^ pfu/ml
• Lesions may be anesthetized with local ice pack prior to injection and/or local anesthetic
• May use four quadrant or fan technique (see Figure 1); may need to avoid necrotic areas and inject locations with viable tumor cells (i.e., periphery)
• Injector should use universal precautions
• Portable ultrasound may be useful if lesion regresses or is not clinically palpable

**TABLE 3:** Tumor volume determination for T-VEC administration.

Lesion size (longest diameter)	T-VEC injection volume
>5 cm	UP to 4 ml
>2.5–5 cm	UP to 2 ml
>1.5–2.5 cm	UP to 1 ml
>0.5–1.5 cm	UP to 0.5 ml
≤0.5 cm	UP to 0.1 ml

Abbreviations: cm, centimeter; ml, milliliters; T-VEC, talimogene laherparepvec.

**TABLE 4:** Considerations for patient management after T-VEC injection.

• Site should be wiped with alcohol prior to injection and after bandage is placed
• Sites of injection should be covered with dry gauze and virus impenetrable occlusive dressing (e.g., Tegaderm dressing)
• Biohazard waste receptacles for dry waste and needles should be in the treatment room
• Bandages should be maintained for 5–7 days
• Patient should be given extra bandages in case replacement is needed and provided with education on how to manage (e.g., hand washing, gloves, proper disposal of waste)
• Acyclovir can be used for accidental exposure
• Pregnant woman and immunosuppressed individuals should avoid direct contact with T-VEC-injected patients for 7 days

Subset analyses have suggested that certain populations may receive especial benefit from T-VEC, including melanomas of the head and neck, older patients who may also have other co-morbid conditions, and transplant recipients. Melanoma metastasis to the central nervous system (CNS) remains a significant clinical challenge and recent evidence that oncolytic HSV-1 (teserpaturev) has activity in glioblastoma suggests that T-VEC could be considered for treating CNS melanoma. Direct access to the CNS for bimonthly injections remains a logistical challenge but further clinical studies may be warranted. Further studies are needed to confirm a role for T-VEC in the neoadjuvant setting and for other cancers. While studies of combination treatment with immune checkpoint inhibitors have been contradictory, other therapeutic combinations await clinical validation, including combinations of T-VEC with radiation therapy, targeted therapy, chemotherapy, and adoptive T cell therapy. Predictive biomarkers are also needed and early work suggests that elements of the intracellular anti-viral machinery may be important predictors of OV sensitivity and merit further evaluation. As techniques for single cell genomic analysis have matured, this will provide assays to interrogate tumor cells *in vitro* and *ex vivo,* which should accelerate better patient selection and more rational combination strategies.

Oncolytic viruses represent a new class of cancer therapeutics that have, thus far, resulted in limited approvals for cancer. Newer viruses with more rationally designed transgene payloads, coupled with a better understanding of the underlying biology, should lead to new approvals and best-in-class agents across a range of tumor types and clinical indications. Further studies to explore T-VEC injection of visceral lesions as well as determining the risks and benefits of intravenous delivery are needed. What is established is the tolerable safety profile of T-VEC and other OVs in clinical development with most exhibiting similar low grade and short duration constitutional and local injection site reactions. The safety profile may allow better patient acceptance and expansion of OVs into more immunologically sound combination clinical trials for patients with cancer.
